# Wealthy individual investors and stock markets’ tail risk

**DOI:** 10.1371/journal.pone.0282173

**Published:** 2024-05-20

**Authors:** He Yu, Rong Lu, Hu Yang, Bin Zhang

**Affiliations:** 1 School of Finance, Zhejiang University of Finance and Economics, Hangzhou, China; 2 School of Finance, Shanghai University of Finance and Economics, Shanghai, China; 3 School of Information, Central University of Finance and Economics, Beijing, China; Universidad Veracruzana, MEXICO

## Abstract

This paper employs a unique data set to analyze the trading behavior of wealthy individual investors across Mainland China and their impact on Chinese stock markets’ tail risk. Results show that the wealthy individual investors’ trading behavior can explain Chinese stock markets’ tail risk, and the daily investment portfolios based on the network density of wealthy individual investors have significant excess returns. This paper also investigates the determinants of wealthy individual investors’ trading behavior with the social network method and the spatial econometric model, and reveals that wealthy individuals benefit from the spillover effect of their trading behavior through the investor networks. The results of this paper not only reveal micro evidence for the formation mechanism of asset prices, but also provide insight into the behavior of wealthy individual investors.

## 1. Introduction

Wealthy individual investors’ behavior and influence on financial markets are important issues concerned by scholars and the other types of investors in the financial markets. This paper employs a unique data set to analyze the trading behavior of 128,960 individual investors whose stocks’ market value is over CNY 10 million, as the top 5‰ wealthiest individual investors across Mainland China. Through establishing the trading networks of wealthy individual investors, this paper examines the influence mechanism of potential correlated transactions of wealthy individual investors on the tail risk of China’s stock market. Conclusions in this paper deepen the understanding of the price formation mechanism of China’s capital market, and provide a reference for exploring wealthy individual investors’ behavior.

Firstly, this paper focuses on stock markets’ tail risk since the stability of the capital market is of great significance to increase residents’ property income. The 17th National Congress of the Communist Party of China for the first time proposed the policy of "creating conditions for more people to have property income". The 18th National Congress of the Communist Party of China more clearly come up with the goal of "increasing residents’ property income through multiple channels". As we know the investment in multi-level capital markets is an important way for Chinese residents to increase their property income. The tail risk has strong predictive power for aggregate market returns(Kelly and Jiang, 2014) [1]. However, the tail risk of China’s stock market is among the highest in the world. A-share investors in China made an average profit of 29,400 yuan in 2017, while 70% of investors lost money (Oriental Wealth Network, 2017) [2]. Ahead of the 2018 Lunar New Year, China’s A-share market plunged, while market fears play an important role in understanding the return predictability (Bollerslev and Todorov, 2015) [3] and greatly affected the investors’ return. In this way, the fluctuations in the capital market price tickle the nerves of investors. A healthy and stable capital market promotes the growth of residents’ property income.

Secondly, the trading resonance behavior of wealthy individual investors affects the stability of the capital market. On the one hand, Shi (2017) [4] pointed out that wealthy individual investors may make loose or logically concerted moves in popular stocks, encouraging huge fluctuations in the stock market. On the other hand, China’s stock market differs from the developed capital market in that small and medium investors have high turnover rates and their trading decisions are easy to be guided by the main funds of wealthy individual investors. Literature also found that institutional investors also show herd behavior, while wealthy individual investors as information insiders make more aggressive trading decisions (Chen et al., 2009; Li et al., 2017) [5, 6]. Therefore, wealthy individual investors’ behavior and their potential correlated transactions both directly and indirectly affect the efficiency and stability of capital markets.

This paper discovers that there exists a trading resonance among some wealthy individual investors, which directly affects the tail risk of the markets. According to the definition of connected investors by Jiang et al. (2013) [7] and Ozsoylev et al (2014) [8], and combined with the characteristics of wealthy individual investors’ behavior, this paper defines each pair of investors as connected investors if and only if these two investors submit transaction orders of the same stock within a time window of one day at least 3 times over a half year. First, the trading of the same stock on the same day may not result from information exchange, but from the similarity of trading strategies, and this does not affect the effect of similar trading actions on the market’s tail risk. Second, wealthy individual investors often appear in groups in individual stocks. The more similar trading decisions there are, the closer the connection between these two investors is. Third, unlike ordinary investors with the same trading purpose submit trading orders in the same directions, connected wealthy individual investors may sometimes submit trading orders of opposite directions. Therefore, the affiliated connection standard set in this paper does not require two wealthy individual investors to trade individual stocks in the same direction.

According to the definition of investors’ connection, this paper uses the account level transaction data in the first half year of 2017 to calculate the networks of experienced wealthy individual investors. Firstly, the empirical wealthy individual investors’ network has been proved to be quite stable over time with either the full sample data or the sub-sample data. Secondly, the paper studies the influence of empirical wealthy individual investors’ network characteristics and investor characteristics on the stock’s tail risk. Thirdly, samples are divided into two intervals in robustness tests. For the first interval, the empirical wealthy individual investors’ network is constructed. For the second interval, this paper tests the influence of the prior empirical wealthy individual investors’ network on the individual stocks’ tail risk. It is found that the network density of wealthy individual investors has a positive impact on individual stocks’ tail risk, and the investment strategy constructed based on the density of wealthy individual networks can obtain an excess yield of 65%.

This paper further studies the formation mechanism of wealthy individual investors’ behavior affecting the stock market’s tail risk. Results show that the wealthy individual investors in the center of the investor networks gain higher excess returns, while there exists transaction resonance in wealthy individual investors’ networks. The results suggest that investors have an incentive to influence other investors by trading contagion, thus improving their gains. In the process, the trading contagion of wealthy investors increases the stock markets’ tail risk.

Compared with the existing literature, this paper may contribute in the following three aspects. First, the investigation of similar trading actions of wealthy individual investors expands the research on the trading resonance and tail risk in the capital market. Considering that existing research on the tail risk mainly focuses on the system risk caused by contagion between companies or banks, this paper, based on the wealthy individual investors’ account data provided by a national securities firm, investigates the contagion channels between micro individual investors, providing a new perspective for the study of stock markets’ tail risk.

Second, this paper broadens the cognition of wealthy individual investors. The existing research on investors’ behavior mainly focuses on institutional investors and retail investors, but pays less attention to wealthy individual investors, especially their potential trading connection. In this paper, the unique account level data of the top 5‰ wealthiest individual investors are used, which can more accurately study specific investors than the time-based high-frequency data which is investors’ trading aggregate data. This paper studies the impact of wealthy individual investors’ behavior from the perspective of network structures, and analyzes the interaction of wealthy individual investors’ income from the transaction contagion path, which can expand the research on individual investors’ behavior.

## 2. Literature

### 2.1 Tail risk in capital markets

The tail risk in China’s stock market is considerably higher than the same risk in the other capital markets. One of the critical characteristics that distinguishes China’s stock market from mature capital markets is that China’s stock market is dominated by retail investors who are less rational than the other types of investors, which leads to drastic fluctuation in stock price and apparent deviation of stock prices from intrinsic value. Therefore, many researchers study the tail risk of China’s capital market from the perspective of behavioral finance.

The perspective of heterogeneous investors’ behavior reveals micro reasons for tail risks in stock markets. Chen et al. (2017) [9] found herd behavior among Chinese individual investors and institutional investors, believing it has led to the tail risk in China’s stock market. Ng and Wu (2007) [10] proposed that only the trading activities of institutional investors and wealthy individual investors affect the volatility of China’s stock market. Giannini et al. (2019) [11] discovered that the convergence and divergence of opinion have significant effects on trading volume and return. With the convergence of opinion, earnings announcement returns are lower; otherwise, the returns are higher. Li and Jiang (2022) [12] proposed that well-connected institutions increase stock price crash risk, and they are partially attributed to reduced accounting conservatism and investor herding. Kostopoulos et al. (2022) [13] used individual investors’ trading records data and discovered that increasing in ambiguity is related to higher frequency in investor activities. Nicolas (2022) [14] estimated a model of herding behavior on social networks and pointed out that social interactions explain investor sentiment and assets’ volatility, especially explaining highly volatile assets. Gong and Diao (2022) [15] showed that a high variance of investor networks’ degree distribution might cause market instability.

However, wealthy individual investors appear in groups in popular stocks, which may lead to information exchange, connected trading and tail risk (Shi, 2017) [4], but are not analyzed further enough. Shi and Chen (2004) [16] and Chen et al. (2013) [17] found that wealthy individual investors are the most informed investors and they affect the tail risk of stock markets; while retail investors are noise traders providing liquidity, and institutional investors show herd behavior. Yang et al. (2022) [18] proposed that the proportion of informed investors’ wealth affect the speed of information diffusion, and stocks with faster information diffusion speed gain higher time-series momentum returns, especially under short holding period strategies.

### 2.2 Trading behavior of wealthy individual investors

The earliest research on wealthy individual investors in China came from the Shanghai Stock Exchange (Shi and Chen, 2004; Xiao and Wang, 2010) [16, 19]. Later, some scholars, using investors’ account data from China’s stock exchanges and securities business departments, further pointed out that wealthy individual investors have an important impact on the capital market in a way that is different from other types of investors. This paper discusses the research status of wealthy individual investors from three aspects: the definition, behavior characteristics, and their impacts.

#### 2.2.1 The definition of wealthy individual investors

The basic judgment criterion for wealthy individual investors is whether their trading activities have a substantial potential impact on the market (Sacks and Blankenship, 2012) [20]. The Securities and Exchange Commission (SEC) defines the large trader as an individual or institution that invests in "NMS securities" (all securities traded on national exchanges, including stocks and options) with a daily volume of at least 2 million shares or $20 million a day or at least 20 million shares or $200 million a month (SEC, 2011) [21]. Ng and Wu (2007) [10] and Chen et al. (2013) [17] divided the participants in China’s stock market into small and medium investors, wealthy individual investors, funds, and other institutions referring to the investor structure classification standard commonly used by Shanghai and Shenzhen stock exchanges and positioned wealthy individual investors as individual investors with assets of more than 1 million yuan. Li et al. (2017) [5] defined investors owning more than CNY 1 million portfolio value as wealthy individual investors, accounting for about 0.5% of all individual investors in the market.

But overall wealthy individual investors are the investors that academic research ignores. Existing literature mainly classifies investors in two ways. First, investors are divided into informed investors and noise traders according to their information advantages (Kyle, 1985) [22]. Second, investors are divided into institutional investors and individual investors according to their social attributes (Brunnermeier and Nagel, 2004; Bendavid et al., 2012; Cuthbertson et al., 2016) [23–25]. Some studies combine wealthy individual investors with institutional investors together for analysis. For example, based on the framework of game analysis, Xiao and Tian (2002) [26] studied the pricing strategies of wealthy individual investors, however, they are actually discussed investors who are opposite to retail investors and mostly of them are institutional investors. In all, all those classification methods do not take into account the transaction attributes of investors, which results in insufficient research on wealthy individual investors.

#### 2.2.2 Behavior and influence of wealthy individual investors

Wealthy individual investors with abundant funds have capital and information advantages but are not subject to much strict supervision, so they are different from other types of investors, such as small and medium investors, public or private funds. Coval et al. (2005) [27] found that the returns of individual investors were consistent, and therefore proposed that some investors had advantages on selecting stocks. Feng and Seasholes (2005) [28] pointed out that investors’ investment sophistication and trading experience mitigate behavioral biases, and wealthy individual investors are more sophisticated than other types of investors. Shi and Chen (2004) [16] found that wealthy individual investors were the most informed investors in the stock market. Ng and Wu (2006) [29] showed that wealthy individuals prefer stocks with high liquidity and volatility, greater state-ownership, high growth potential, and strong past performance. Ng and Wu (2007) [10] found that a small group of wealthy Chinese individuals had similar trading choices with institutional investors when buying stocks, which showed they are momentum investors; while they had similar trading behavior with small and medium individual investors when selling stocks, showing the characteristics of contrarian investors. Chen et al. (2013) [17] discovered that small and medium investors in the Chinese market are reverse traders who "chase down and sell up", funds investors are inertial traders who "chase up and sell down", and wealthy individual investors are groups with relatively strong investment ability. Tekçe and Yılmaz (2015) [30] discovered that investors with a higher portfolio value are less likely to exhibit overconfidence. Tekçe et al. (2016) [31] investigated behavioral biases among individual stock investors and found that investors with high portfolio values are more likely to be subjected to disposition effect and representativeness heuristic. Li et al. (2017) [5] utilized the account data of individual investors in China’s stock market and found that wealthy individual investors in the top 0.5% of wealth can obtain significantly higher returns than other investors. By the methodology of event study, they also proposed that the high returns of wealthy individual investors can be attributed to their information network advantages to a certain extent. Carpio et al. (2021) [32] revealed that wealthy investors are more likely to participate in foreign stock markets, but as investor wealth increases, the portfolio share invested in foreign equities tends to decrease. Bender et al. (2022) [33] discovered that wealthy investors’ equity share is most affected by professional advice, time until retirement, personal experiences, rare disaster risk, and health risk. Baeckström et al. (2021) [34] discovered that financial advisors tend to recommend wealthy investors with higher-risk portfolios. Bui et al.(2022) [35] pointed out that wealthy investors take extra risks and earn significantly higher returns in the stock market. Therefore, it is seen that there are different heterogeneous behavior among individual investors and wealthy individual investors have unique transaction behavior characteristics.

The behavior of wealthy individual investors affects the price volatility of capital markets. Wealthy individual investors are found to appear in groups in popular stocks, contributing to the tail risk of the stock market (Shi, 2017) [4]. Whether there is a loose or logically concerted action among these wealthy individual investors has always been the focus of regulators’ attention (Jiang et al., 2013; Shi, 2017) [4, 7]. First, the behavior of wealthy individual investors directly affects the stock market price. Ng and Wu (2007) [10] found that the behavior of wealthy individual investors can cause stock market fluctuations. Xiao and Wang(2010) [19] used the trading data of investor accounts in the Shanghai Stock Exchange to study the relationship between various investors’ trading strategies and the stock market crash, and they found that wealthy individual investors had an important influence on the tail risk of stock prices. Second, wealthy individual investors may have the potential for manipulating stock markets. Li et al. (2017) [5] found that wealthy individual investors with information advantages may manipulate stock prices when listed companies announce information. Third, some wealthy individual investors use non-sunshine private equity funds as their trading accounts, and studies showed that some hedge funds manipulate stock price. Bendavid et al. (2012) [24] pointed out that in order to perform better in peer rankings, some hedge funds manipulated stock prices during important reporting periods. Hence, no good ways have been proposed to identify wealthy individual investors from investors, and researches about identifying wealthy individual investors from all investors and studying their potential impacts as a group still have a lot of work to do.

### 2.3 Literature review

Through the review of the research literature, the following points can be concluded. First, researches on the influence of heterogeneous investors on the stock market’s tail risk mostly focus on quarterly or monthly impacts, but less on detailed analysis of the influence of heterogeneous investors’ behavior on short-term sharp stock fluctuations. Second, the research on investors’ behavior is mainly limited to retail investors and institutional investors, without in-depth discussion on wealthy individual investors who have a special influence on the market, especially investors with portfolio values greater than 99.5‰. Third, even some research focus on the direct effects of wealthy individual investors, they do not explore their combined impacts as a group, that is, the connections among wealthy individual investors and their comprehensive impacts on capital markets.

Based on the account data of wealthy individual investors, this paper proposes a method to identify the relationship between the behavior of wealthy individual investors and the stock markets’ tail risk, which is helpful to expand the research on the formation of short-term asset prices and the tail risk, enrich the theory of individual investors’ behavior, and broaden the perspective of supervising wealthy individual investors in practice.

## 3. Research design

### 3.1 Samples and data sources

Individual investors with portfolio values of more than CNY 10 million are defined as wealthy individual investors in this paper. The database utilized in this paper comes from a securities firm and the data covers all wealthy individual investors’ daily transaction information from January 2017 to June 2017, including variables of stock code, trading date, shareholder code, one code number, investor type, transaction buying amount, transaction buying average price, transaction selling amount and transaction selling average price.

The analysis of the behavior of wealthy individual investors is based on all the trading date of the listed companies of a securities firm, while the analysis of individual stocks excludes data on the stocks of companies that have been listed for less than one year, suspended trading, ST stocks, and banking financial stocks. In this paper, the tail of 1% level has been deleted and the financial data of listed companies come from the CSMAR database.

### 3.2 Networks of wealthy individual investors

#### 3.1.1 Overall network of wealthy individual investors

Wealthy investors’ overall network is constructed by nodes and lines, where a node stands for wealthy individual investor and a line between two nodes means there exists some connections between two investors. Based on the research ideas of Jiang et al. (2013) [7] and Ozsoylev et al. (2014) [8], this paper describes the potential resonance correlation between wealthy individual investors based on the similarity of investors’ trading behavior.

Combined with related literature and behavior characteristics of wealthy individual investors, this paper believes that two wealthy individual investors with potential resonance correlation may trade the same stock on the same day, but maybe in the opposite trading direction for the sake of impacting stock prices. Two wealthy individual investors trading the same stock on the same day may happen by chance instead of trading on purpose. This paper assumes that the more similar trading behavior, the closer the resonance correlation between wealthy individual investors. The similar trading behavior of wealthy individual investors may be due to their direct information exchange in the same trading room, or their similar trading strategies. However, trading resonance, regardless of its reasons, has more influence on individual stocks than random trading.

In China’s stock market, wealthy individual investors often trade the same individual stock at similar time period, indicating that there may exist potential connections among wealthy individual investors (Shi, 2017) [4]. This paper evaluates the existence of resonance between two wealthy individual investors in the empirical analysis by identifying if they have traded the same stock on the same day and if these similar trading occur for more than three times. In the robustness test, this paper also establishes two investors’ trading resonance on other criteria, but the key idea is that two investors trade similarly. Based on the determination of resonance correlation between wealthy individual investors, this paper constructs a wealthy individual investors’ network and represents it by a matrix. Assume that the number of wealthy individual investors’ accounts is N, and the network matrix is N×N square matrix *M*_*N*×N_. If wealthy individual investors i and j have the resonance correlation defined in this paper, they will be identified as resonant wealthy individual investors, and the corresponding element M (i,j) = 1 in matrix M; otherwise, M (i, j) = 0.

#### 3.1.2 Dynamic sub-networks of wealthy individual investors

Based on the overall network of wealthy individual investors and the information about who trade in each individual stock, this paper extracts wealthy individual investors’ dynamic sub-networks from their overall network. To construct a network for one company at a certain time span, the first step is identifying the wealthy investors who trade the company’s stocks in a certain time and set them as nodes in the networks; the second step is extract the lines among the nodes according to the links show in the overall network which is built in section 3.1.1. As it is know, if investors’ relationships are extracted from a single company’s stocks or in a certain short time span, important resonance correlations may be missing. However, extracting sub-networks from the overall network can absorb more accurate information, as the overall network spans all companies’ stocks and the entire trading time.

### 3.3 Model and variables

Wealthy individual investors own the most fortune in the financial market, and their resonance behavior may be a reason for stocks’ tail risk. This paper studies the impact of wealthy investors’ resonance trading on the tail risk of capital markets. Three indexes are used to measure stocks’ tail risk, that is stock prices’ daily amplitude and dummy indicators of whether the price rises or falls more than 2% within 3 minutes. Besides, the mean of daily amplitude in a week and the number of days that prices increase or decline more than 2% in a week are used as the proxy variable of weekly price fluctuation.

The wealthy individual investors’ network density measures of the closeness of the correlation between wealthy individual investors in stocks. The network density of stock i is defined as the ratio of the actual number of connected edges between nodes in the network T(i) of stock i to the maximum number of possible edges. The greater network density of wealthy individual investors in individual stocks represents a greater possibility of information diffusion and a faster trading resonance of wealthy individual investors. The network density of stock i can be expressed as:

$${dStkdense}_{i}=2E_{i}/k_{i}(k_{i}-1)$$ (1)

Where, *E*_*i*_ is the actual number of connected edges in the stock network T(i); *k*_*i*_ is the number of elements (wealthy individual investors) in the stock network T(i); *k*_*i*_(*k*_*i*_−1) is the maximum number of edges that *k*_*i*_ wealthy individual investors connect.

This paper uses the Logit model to analyze whether there are abnormal fluctuations in individual stocks, and utilizes the OLS model to analyze the abnormal fluctuations of daily and weekly amplitude. The basic model of the impact of wealthy individual investors’ trading behavior on stock price fluctuations is:

$${Vol}_{i,t}=\alpha +\beta_{1}{dStkdense}_{i,t-1}+\beta_{2}{Vol}_{i,t-1}+\gamma X_{i,t-1}+{Industry}_{i}{+\varepsilon }_{i,t}$$ (2)

Where, *i* and *t* stand for companies and time respectively; *Vol* represents the tail risks; *dStkdense* stands for the network density of wealthy individual investors; *X* is the control variable group, referring to Chen et al. (2001) [36] and Chen et al. (2017) [9]. To avoid the endogenous problem of mutual causality, except for the market risk premium Rm_Rf, the explaining variable and all other control variables are lagged for one period. Industry_i_ signifies the industry fixed effect is taken into consideration. The description of relevant variables is shown in **Table 1**.

**Table 1:** Description of variables.

Name	Code	Description
Explained variables		
Daily volatility	*dVol_Ampli*	Daily amplitude
Daily abnormal rising fluctuation	*dVol_Upret*	0–1 variable, whether it increases by more than 2% in three minutes
Daily abnormal falling fluctuation	*dVol_Downret*	0–1 variable, whether it decreases by more than 2% in three minutes
Weekly volatility	*wVol_Ampli*	Average of daily amplitudes in a week
Weekly abnormal rising fluctuation	*wVol_Upret*	Number of days with abnormal increase in a week
Weekly abnormal falling fluctuation	*wVol_Downret*	Number of days with abnormal decrease in a week
Explaining variables		
Daily network density	*dStkdense*	The ratio of the actual number of connected edges between wealthy individual investors in a stock network to the maximum number of possible edges
Daily quantity of wealthy individual investors	*dHbigcnt*	Logarithm of daily quantity of wealthy individual investors
Weekly network density	*wStkdense*	The ratio of the actual number of connected edges between wealthy individual investors in a stock network to the maximum number of possible edges
Weekly quantity of wealthy individual investors	*wHbigcnt*	Logarithm of weekly quantity of wealthy individual investors
Control variables		
Market risk premium	*Rm_Rf*	Difference between daily market portfolio and risk-free return
Illiquidity	*Amihud*	Price volatility caused by unit trading volume, expressed by the cumulative average of daily rate of return and daily transaction volume over a period of time
Circulation market value	*Lnsize*	Logarithm of circulation market value
Book to market ratio	*Bm*	The ratio between the sum of owner’s equity and the circulation market value of the stock in the previous year
Stock price	*Price*	Logarithm of stocks closing price plus 1
Listing years	*Age*	Logarithm of the number of years of listing plus 1
Asset liability ratio	*Lev*	Dividing the total liabilities of the listed company by the total assets
Net interest rate of total assets	*Roa*	Expressed by the ratio of net profits to total assets, the calculation formula is 2 × (net profit + profits and losses of minority shareholders) / (total assets at the beginning of the period + total assets at the end of the period)
Agency cost	*Ac*	Expressed as the ratio of administrative expenses to total operating income
Expense rate	*Er*	Expense rate of listed companies during sales
Accounts receivable turnover	*Rtr*	Total operating income / [(total assets at the beginning of the period + total assets at the end of the period) / 2]
Profit margin of operating activities	*Oiebt*	Expressed as the ratio of net profits from operating activities to total profits
The sum of the shareholding ratio of the top ten shareholders	*Sum10*	The sum of the shareholding ratio of the top ten shareholders of a listed company
Difference in shareholding ratios of the top two major shareholders	*Dis12*	Difference in shareholding ratios between the top 1 major shareholder and the top 2 major shareholder of a listed company

## 4. Empirical results and analysis

### 4.1 Descriptive statistics

Table 2 reports descriptive statistics of the main variables of individual stocks. As can be seen from Table 2, the average intra-day amplitude of individual stocks in the sample period is 2.89%, and 9.28% of the stocks’ prices increase more than 2% in 3 minutes, while 6.18% of the stocks’ prices decrease more than 2% in 3 minutes. The weekly data shows that the average intra-day amplitude in a week is 2.88% and the average abnormal fluctuation of stocks increasing happen 0.42 days per week and abnormal decreasing occur 0.28 days per week. The above data shows that individual stocks are more vulnerable to get upward price attack, which is quite consistent with the behavioral characteristic of wealthy individual investors.

**Table 2:** Descriptive statistics.

	Variables	N	Mean	Std. Dev.	Min	Median	Max
Explained variables	*dVol_Ampli*	113347	0.0289	0.0192	0.0000	0.0233	0.1438
	*dVol_Upret*	113347	0.0928	0.2901	0.0000	0.0000	1.0000
	*dVol_Downret*	113347	0.0618	0.2409	0.0000	0.0000	1.0000
	*wVol_Ampli*	24088	0.0288	0.0140	0.0062	0.0255	0.0953
	*wVol_Upret*	24088	0.4174	0.8247	0.0000	0.0000	5.0000
	*wVol_Downret*	24088	0.2797	0.6512	0.0000	0.0000	5.0000
Explaining variables	*dStkdense*	113347	0.6087	0.1330	0.1667	0.6105	1.0000
	*dHbigcnt*	113347	3.3863	0.8680	1.0986	3.2958	6.1203
	*wStkdense*	24088	0.3881	0.1275	0.1238	0.3714	1.0000
	*wHbigcnt*	24088	4.4135	0.8444	1.0986	4.3694	6.9460
Control variables	*Rm_Rf*	24088	0.0010	0.0130	-0.0232	-0.0002	0.0228
	*Amihud*	24088	0.0197	0.0197	0.0000	0.0137	0.1700
	*Lnsize*	24088	22.8834	0.9672	20.9006	22.7321	26.1289
	*Bm*	24088	0.5516	0.4031	0.0157	0.4474	2.4336
	*Price*	24088	2.5974	0.5849	1.3829	2.5595	4.1794
	*Age*	24088	2.5657	0.6912	0.7301	2.8359	3.2523
	*Lev*	24088	0.4796	0.2082	0.0728	0.4744	0.9343
	*Roa*	24088	0.0365	0.0498	-0.1982	0.0294	0.1921
	*Ac*	24088	0.1050	0.1098	0.0088	0.0748	0.7341
	*Er*	24088	0.1934	0.1644	0.0218	0.1472	1.0760
	*Rtr*	24088	0.0192	0.0171	0.0014	0.0147	0.1170
	*Oiebt*	24088	0.1797	2.0971	-15.8502	0.7487	1.4967
	*Lnasset*	24088	22.8217	1.5012	19.5234	22.6165	27.3206
	*Sum10*	24088	0.5868	0.1607	0.2179	0.5880	0.9291
	*Dis12*	24088	0.2604	0.2047	0.0004	0.2192	0.7932

The average stock daily network density is 0.6087, the median is 0.6105, the average stock weekly network density is 0.3881, and the median is 0.3714. The mean value is close to the median value, indicating that the distribution of stock network density is relatively equal. Each investor’s multiple transactions of the same stock in a day are recorded as one transaction. On average 29.5 (*e*^3.386^) wealthy individual investors hold the same companies’ stock per day, and the weekly number of wealthy investors in each stock is 82.55 (*e*^4.414^), indicating that the average weekly flow of wealthy individual investors is relatively large, and the current ratio is as high as 279%(82.55/29.5).

### 4.2 Regression results and analysis

According to other control variables, the lag term coefficient of stock fluctuation is significantly positive, indicating that the fluctuation of stock is persistent in time series. The tail risk of the stock prices is higher with the lower liquidity, the lower the book value ratio, the longer the listing period, the lower profitability, and the relatively concentrated equity.

**Table 3** shows the empirical results of the impact of wealthy individual investors’ trading on the daily rise and fall of stocks. Results show that the network density of wealthy investors is positively correlated with the tail risks of financial markets, that is, the rise of the resonance degree of wealthy individual investors cause the rise of abnormal fluctuation of individual stocks. Column (1) indicates that if the daily network density of stocks increases by 1 unit, the daily amplitude of individual stocks increases by 1.04%. Column (2) (3) shows the impact of the network density of wealthy individual investors on the abnormal rise and abnormal decline of stocks. For each 1-unit increase in stock daily network density, the probability of abnormal stock rise increases by 95.81%, and the probability of abnormal stock decline increases by 25.88%. When the trading resonance of wealthy individual investors is high, it would cause other types of investors to follow their trading action and lead to the sharp rise and fall of individual stocks, which is always seen in stock speculation or manipulation events. Besides, increasing the number of wealthy individual investors significantly increases stock price volatility.

**Table 3:** Impact of wealthy individual investors’ network density on daily stock prices’ tail risk.

Variables	OLS	LOGIT	LOGIT
	(1)	(2)	(3)
	*dVol_Ampli*	*dVol_Upret*	*dVol_Downret*
*dStkdense*	0.0104***	0.9581***	0.2588**
	(26.21)	(10.53)	(2.34)
*dHbigcnt*	0.0037***	0.8104***	0.8784***
	(34.48)	(40.76)	(36.94)
*dVol_t0*	0.3295***	1.0373***	1.1109***
	(78.12)	(36.49)	(29.93)
*Rm_Rf*	-0.4408***	-25.7979***	-98.4506***
	(-45.11)	(-12.85)	(-35.35)
*Amihud*	0.0161***	7.5094***	12.0225***
	(5.70)	(17.59)	(24.76)
*Lnsize*	-0.0038***	-0.7879***	-0.8429***
	(-43.13)	(-37.91)	(-33.71)
*Bm*	-0.0022***	-0.5420***	-0.5174***
	(-13.63)	(-13.67)	(-10.62)
*Price*	0.0009***	-0.1465***	-0.0285
	(7.45)	(-5.24)	(-0.84)
*Age*	0.0007***	0.1724***	0.1881***
	(7.32)	(7.80)	(7.03)
*Lev*	0.0006	-0.0932	-0.0636
	(1.36)	(-1.13)	(-0.64)
*Roa*	-0.0155***	-2.9018***	-3.1623***
	(-10.55)	(-10.07)	(-9.23)
*Ac*	0.0034***	-0.2395	-0.1837
	(2.95)	(-0.96)	(-0.62)
*Er*	-0.0006	0.3522**	0.3096
	(-0.86)	(2.24)	(1.64)
*Rtr*	0.0019	-0.1849	0.0970
	(0.44)	(-0.23)	(0.10)
*Oiebt*	-0.0002***	-0.0241***	-0.0243***
	(-5.71)	(-5.11)	(-4.20)
*Sum10*	0.0080***	2.0935***	2.1498***
	(16.63)	(19.60)	(16.56)
*Dis12*	-0.0000	-0.1841**	-0.1500*
	(-0.06)	(-2.56)	(-1.71)
*Constant*	0.0774***	10.7018***	11.1910***
	(41.94)	(23.80)	(20.55)
Industry FE	yes	yes	yes
N	113,347	113,347	113,347
*R*^2^*(*Pseudo *R*^2^*)*	0.25	0.1348	0.1669

Note: *dVol_t0* is the lag term of the corresponding daily fluctuation, that is, *dVol_Ampli_t0、dVol_Upret_t0、dVol_Downret_t0* respectively. Data in parentheses is the t value

***, ** and * indicate significant at the 1%, 5% and 10% levels, respectively. The last data in Column (1) is *R*^2^ and in Column (2) (3) are Pseudo *R*^2^.

According to other control variables, the lag term coefficient of stock fluctuation is significantly positive, indicating that the fluctuation of stock is persistent in time series. The tail risk of the stock prices is higher with the lower liquidity, the lower the book value ratio, the longer the listing period, the lower profitability, and the relatively concentrated equity.

This paper further investigates the network density’s impact on tail risks from the weekly perspective. Table 4 shows that similar to the results in According to other control variables, the lag term coefficient of stock fluctuation is significantly positive, indicating that the fluctuation of stock is persistent in time series. The tail risk of the stock prices is higher with the lower liquidity, the lower the book value ratio, the longer the listing period, the lower profitability, and the relatively concentrated equity.

**Table 4:** Impact of wealthy individual investors’ network density on weekly stock prices’ tail risk.

Variables	(1)	(2)	(3)
	*wVol_Ampli*	*wVol_Upret*	*wVol_Downret*
*wStkdense*	0.0090***	0.6053***	0.2525***
	(13.69)	(14.16)	(7.56)
*wHbigcnt*	0.0029***	0.1463***	0.1549***
	(16.28)	(14.77)	(19.59)
*wVol_t0*	0.4015***	0.2656***	0.1867***
	(43.46)	(26.60)	(17.31)
*Rm_Rf*	-0.0916***	-3.1090***	-7.3499***
	(-15.94)	(-7.75)	(-21.98)
*Amihud*	0.0095	1.6204***	1.4813***
	(1.40)	(4.09)	(4.56)
*Lnsize*	-0.0031***	-0.1668***	-0.1532***
	(-23.46)	(-19.58)	(-22.10)
*Bm*	-0.0018***	-0.1402***	-0.1093***
	(-7.63)	(-8.82)	(-8.35)
*Price*	0.0011***	-0.0013	0.0258**
	(6.20)	(-0.11)	(2.57)
*Age*	0.0006***	0.0342***	0.0290***
	(4.34)	(3.48)	(3.66)
*Lev*	0.0006	-0.0236	-0.0209
	(1.00)	(-0.57)	(-0.62)
*Roa*	-0.0152***	-1.0960***	-0.9295***
	(-7.29)	(-7.52)	(-7.82)
*Ac*	0.0019	-0.0102	0.0297
	(1.18)	(-0.10)	(0.33)
*Er*	0.0005	0.1086*	0.0657
	(0.46)	(1.68)	(1.23)
*Rtr*	0.0047	0.5279	0.5390
	(0.74)	(1.23)	(1.47)
*Oiebt*	-0.0001***	-0.0135***	-0.0094***
	(-3.17)	(-3.69)	(-3.29)
*Sum10*	0.0069***	0.5075***	0.4714***
	(9.99)	(10.90)	(12.66)
*Dis12*	-0.0002	-0.0893***	-0.0576**
	(-0.50)	(-2.90)	(-2.35)
*Constant*	0.0637***	2.9029***	2.5927***
	(22.81)	(16.13)	(17.41)
Industry	yes	yes	Yes
N	24,088	24,088	24,088
*R* ^2^	0.379	0.196	0.170

Note: *wVol_t0* is the lag term of the corresponding daily fluctuation, that is, *wVol_Ampli_t0、wVol_Upret_t0、wVol_Downret_t0* respectively. Data in parentheses is the t value

***, ** and * indicate significant at the 1%, 5% and 10% levels, respectively.

**Table 3**, the network density of wealthy individuals is related to stocks’ sharp surge or slump, but the impact is relatively reduced. An increase in weekly resonance intensity by one unit will lead to a 0.9% increase in weekly average amplitude, 0.61 days for abnormal increases with more than 2% within 3 minutes, and 0.25 days with abnormal declines of more than 2% within 3 minutes.

### 4.3 Endogenous and robustness test

The potential problem is that high tail risk stocks’ networks have the characteristics of high network density is only because the wealthy individual investors jointly choose the stocks with high tail risk for investment. Therefore, the sample is divided in two stages to solve the problem of endogenous. In the first four months, the data is used to establish investors’ connections and build the overall network. While in the next two months, the data is utilized to build stocks’ sub-networks, calculate the network density and analysis its effects on the tail risk.

Even based on the investors’ historical transaction records to identify investors’ connections and construct networks, the conclusions are still consistent with those of the main test, which means the investors’ connections sustain and endogenous problem can be solved. When wealthy individual investors with resonance correlation appear centrally in stocks, the amplitude of stocks increases, and the stock price is very likely to rise or fall within 2 minutes.

Other robustness tests include different methods to judge the connections among investors, use various time period’s data as datasets, and other related similar explained variables to regress. All these tests verify the conclusions of this paper.

## 5. Mechanism analysis

### 5.1 Theory models and analysis

This paper use three models to analysis the influence mechanism of wealthy individual investors’ network density on the tail risk in capital market. The first model tests the existence of wealthy individual investors’ networks, that is, whether wealthy individual investors are affected by the connected investors and follow them to conduct concurrent transactions and resonance. The second model studies whether wealthy individual investors gain higher return because of the more central positions in the network. The first model shows the formation process of wealthy investors’ trading resonance, and these actions may expand and conduct stocks’ abnormal fluctuation. The second model shows the transaction motivation of wealthy individual investors to be more involved in the trading network. By then, we get the information of the process of wealthy individual investors’ behavior on capital market’s tail risk. The third model consider the cross term of investors’ average centrality and network density of each company’s dynamic sub-networks, so as to identify if investors’ trading resonance impact stocks’ tail risk through wealthy individual investors’ influence power and behavior contagion effect.

#### 5.1.1 Existence and causes of wealthy individual investors’ network

Based on the ideas of Lee, et al. (2010) [37] and Cohen-Cole et al. (2014) [38], by analyzing the characteristics of the wealthy individual investors’ transaction network, this paper investigates the contagion effect of wealthy individual investors’ behavior, quantifies the speed and expansion multiple of the contagion.

The spatial auto-regressive model(SAR) can be used to investigate whether there is transaction resonance among wealthy individual investors. The basic assumption is that trading decisions made by wealthy individual investors in the current period are influenced by the behavior of other wealthy individual investors in the previous period. As the direct result of a wealthy individual investors’ behavior is rate of return r_t_, it is easy to infer that the return is related to the rate of return Wr_t−1_ of its correlated wealthy individual investors in the trading network in the past period. Accordingly, the influence of the wealthy individual investor’ behavior can be regarded as a time lagged and space lagged auto-regressive process:

$$r_{t}=\rho Wr_{t-1}+X\beta +\varepsilon_{t}$$ (3)

By replacing r_t−1_ with $r_{t-1}=\rho Wr_{t-2}+X\beta +\varepsilon_{t-1}$, gradually we get:

$$r_{t}=(I_{n}+\rho W+\rho^{2}W^{2}+\cdots +\rho^{q-1}W^{q-1})X\beta +\rho^{q}W^{q}r_{t-1}+u$$ (4)

When |ρ|< 1 and the value of q is very large, the value of ρ^q^W^q^r_t−1_ becomes small. Then, the observed cross-sectional correlation can be interpreted as a expected return of long-term equilibrium or a result in a steady state:

$$\underset{q\to \infty }{\lim}\;E(r_{t})={\left(I_{n}-\rho W\right)}^{-1}X\beta$$ (5)

According to the analysis of network structure, the estimation coefficient ρ of the above model represents the average correlation of return between one wealthy individual investor and its directly correlated wealthy individual investors, and ρ^2^ describes the correlation of wealthy individual investors when the interval increases by one bit. If the return changes by one unit, the overall change range of wealthy individual investors in the network will be as follows:

$$\emptyset =\frac{1}{1-\rho }$$ (6)

Therefore, the process of investors’ trading decision at different spatial points can be analyzed by SAR model to investigate the existence and contagion effect of wealthy individual investors’ trading network.

The expansion formula of model (3) is:

$$r_{i,k}=\alpha_{0}+\rho \frac{1}{d_{i,k}}\sum_{j=1}^{n_{k}}d_{ij,k}r_{j,k}+\sum_{m=1}^{M}\beta^{m}x_{i,k}^{m}+\varepsilon_{i,k}$$ (7)

Where, i = 1,2,…, n_k_; k = 1,2,⋯,K; $d_{i,k}=\sum_{j=1}^{n_{k}}d_{ij,k}$ represents the weighted sum of wealthy individual investors’ direct connections with the others in the network k, which is defined as a variable, degree, in most network analysis methodologies; $\frac{1}{d_{i,k}}\sum_{j=1}^{n_{k}}d_{ij,k}r_{j,k}$ represents the average return of the correlated investors in the network; $x_{i,k}^{m}$ represents m control variables related to wealthy individual investor or the networkl, including the eigenvector centrality and degree of investor i, as well as the transaction amount, transaction days and the number of stocks invested by the wealthy individual investors; and ε_i,k_ is the random error term.

If the wealthy individual investors’ network is meaningless, ρ = 0; otherwise ρ ≠ 0. A large number of wealthy individual investors are involved in this paper and the large sample theory of spatial econometric model using maximum likelihood estimation parameters still needs to be improved (Chen, 2014) [39], so this paper uses the method proposed by Xiao et al. (2012) [40] to test the transaction resonance behavior among wealthy individual investors. The steps are as follows: 1) Calculate the return vector of investors in wealthy individual investors’ network. 2) Quantify the spatial contagion rate of wealthy individual investor behavior. Corresponding to each wealthy individual investor in the dynamic network, this paper calculates the overall average rate of return of its correlated wealthy individual investor, and the interaction coefficient ρ to obtain the spatial contagion speed. 3) In the process of data generation represented by the spatial econometric model, the infection path and the overall impact range of the impact in the wealthy individual investors’ network φ = 1/(1-ρ) are analyzed.

#### 5.1.2 Wealthy individual investors’ return from their network transactions

As the above model has theoretically simulated and empirically explained the reason why transaction networks can describe wealthy individual investors’ trading resonance and its impact, this section adopts the social network method to study the relationship of wealthy individual investors’ return and the network positions, which shows wealthy individual investors’ motivation to conduct trading resonance. According to the research ideas of Ozsoylev et al. (2014) [8], Chung et al.(2018) [41] and He et al.(2022) [42], the basic model is:

$${Ret}_{i}=\alpha_{1}+\beta_{1}{Vecdu}_{i}+\gamma_{1}{Lnd}_{i}+\sum_{j}\theta_{j}{Control}_{i,j}+\varepsilon_{i}$$ (8)

Where *Ret*_*i*_ represents the return or excess return of the investor i; *Vecdu*_*i*_, *Lnd*_i_ represent the investor i’s eigenvector centrality and logarithmic degree respectively; *Control*_*i*,*j*_ includes Ntrd_ln,NSec_ln,Qtrd_ln which represent natural logarithms of transaction times, security quantities, and transaction amount of the investor i during the sample period.

#### 5.1.3 The regulation effect of the investor centrality

As is shown in model(8), if a wealthy individual investor with higher network centrality has more connections with other investors and also has a stronger influence on them, it is reasonable to deduce that the investors in individual stock’s sub-network with higher average centrality would have more influence power on the relationship between network density and stocks’ risk tails. This paper builds model(9) by adding the variable of average centrality of sub-networks’ investors (*Vecdu*) and the variable of a cross term (*dStkdense*×*Vecdu*) to model(2).

$${Vol}_{i,t}=\alpha +\beta_{1}{dStkdense}_{i,t-1}+\beta_{2}{Vecdu}_{i,t-1}+\beta_{3}{dStkdense}_{i,t-1}\times {Vecdu}_{i,t-1}+\gamma X_{i,t-1}+{Industry}_{i}{+\varepsilon }_{i,t}$$ (9)

Where, *i* and *t* stand for companies and time respectively; *Vol*_*i*,*t*_ represents the tail risk of stock *i* at time *t*; *dStkdense*_*i*,*t*_ stands for the dynamic sub-networks’ density of stock *i* at time *t*; *Vecdu*_*i*,*t*_ is the average eigenvector centrality for all wealthy individual investors that invest stock *i* at time *t*; *X*_*i*,*t*_ is the control variable group.

### 5.2 Empirical results of the formation mechanism of wealthy individual investors’ trading behavior

#### 5.2.1 Descriptive statistics

**Table 5** reports descriptive statistical results of the 128,960 wealthy individual investors’ transactions, from which it can be seen that when taking the closing price of each stock on the last day of the sample period as the reference point, for each transaction, the arithmetic average rate of return is 1.66%, the arithmetic average excess rate of return is 1.97%, the average weighted rate of return is 0.995%, and the weighted excess rate of return is 1.27%, which exhibiting the excellent investment performance of wealthy individual investors.

**Table 5:** Overall descriptive statistics of wealthy individual investors’ behavior.

Variables		N	Mean	Std. Dev.	Min	Median	Max
arithmetic average return	*ARet*_*i*_	128960	0.017	0.087	-0.924	0.005	1.057
weighted average return	*WRet*_*i*_	128960	0.010	0.099	-0.911	0.002	1.057
arithmetic average excess return	*AExcRet*_*i*_	128960	0.020	0.089	-0.939	0.006	1.052
weighted average excess return	*WExcRet*_*i*_	128960	0.013	0.101	-0.927	0.002	1.099
Eigenvector centrality	*Vecdu*_*i*_	128960	0.001259	0.002484	0	0.000284	0.037706
Logarithmic degree	*Lnd*_*i*_	128960	4.418	2.664	0.000	4.868	11.218
Logarithmic of trading quantity	*Qtrd*_*ln*	128960	15.249	2.385	1.985	15.630	22.378
Logarithmic of trading times	*Ntrd*_ln	128960	2.849	1.512	0.000	2.833	10.196
Logarithmic of trading securities	*NSec*_ln	128960	1.800	1.068	0.000	1.792	7.122

A large number of wealthy individual investors are studied in the paper, and all investors’ eigenvector centrality has been normalized, that is, setting the sum of squares of all investors’ eigenvector centrality equal to 1, so the normalized eigenvector centrality of each wealthy individual investor is relatively low, with an average centrality of 0.001259. In addition, the standard deviationof the eigenvector centrality of wealthy individual investors’ network is 0.002484, while the skewness and kurtosis which are not shown in **Table 5** are 4.313 and 31.157 respectively, indicating that there are significant differences in the positions of wealthy individual investors in the network, and there are more wealthy individual investors at the center or edges. The logarithmic degree of wealthy individual investors’ network describes the number of direct connections among investors. The natural logarithmic average of the degree value is 4.418, indicating that each wealthy individual investor has 82 correlated wealthy individual investors on average, accounting for 0.06% of the total number of wealthy individual investors.

In the sample period, the average total transaction amount of wealthy individual investors is about CNY 4.2 million (*e*^15.25^), indicating that the turnover rate of the wealthy individual investors with a total position of no less than CNY 10 million is not high. The transaction frequency in this paper refers to the cumulative transaction frequency of the wealthy individual investors in the sample period, and the two-way transaction of the same stock within the same day is seen as two transactions. As can be seen from **错误!未找到引用源。**, if the data is not taken in logarithm, wealthy individual investors trade 17 times (*e*^2.849^) in six stocks (*e*^1.8^) within six months on average.

#### 5.2.2 Empirical results and analysis

Table 6 shows the results of model (7) and model (8). Columns (1) (3) (5) (7) show that eigenvector centrality and logarithmic degree are positively correlated with the return of wealthy individual investors, indicating that the wealthy individual investors’ transaction network constructed in this paper can represent the information network between wealthy individual investors. Wealthy individual investors increase their return in virtue of their correlation with other wealthy individual investors. The result of the correlation between investors’ returns and network logarithmic degree in this paper is different from the result in Ozsoylev et al. (2014) [8] which found that the investors’ degree is negatively correlated with their return. The reason is that the investors studied in Ozsoylev et al. (2014) [8] stand for all types of investors, and most of them do not have information advantages. However, the empirical results of this paper are in line with the characteristics of wealthy individual investors with information advantages and higher information network efficiency, that is, when one wealthy individual investor is correlated to more other wealthy individual investors, its return would rise.

**Table 6:** The return of wealthy individual investors and network centrality.

Variables	arithmetic average return		weighted average return		arithmetic average excess return		weighted average excess return	
	(1)	(2)	(3)	(4)	(5)	(6)	(7)	(8)
*Wr*		0.000307***		0.000360***		0.000296***		0.000270***
		(37.65)		(12.83)		(41.06)		(11.03)
*Vecdu*	2.402***	0.331***	2.054***	0.484***	2.892***	0.525***	2.407***	0.580***
	(22.26)	(3.03)	(16.81)	(3.91)	(25.99)	(4.66)	(19.50)	(4.39)
*Lnd*	0.00436***	0.00375***	0.00222***	0.00269***	0.00488***	0.00431***	0.00254***	0.00339***
	(22.57)	(17.28)	(8.83)	(9.47)	(24.72)	(19.36)	(9.92)	(11.76)
*Qtrd*_*ln*	-0.00323***	-0.00223***	-0.00612***	-0.00489***	-0.00374***	-0.00253***	-0.00685***	-0.00535***
	(-18.89)	(-14.89)	(-30.87)	(-26.42)	(-21.54)	(-16.45)	(-34.07)	(-28.32)
*Ntrd*_*ln*	-0.0288***	-0.0251***	-0.0126***	-0.0102***	-0.0341***	-0.0299***	-0.0156***	-0.0134***
	(-71.34)	(-64.94)	(-24.58)	(-20.26)	(-81.33)	(-74.48)	(-30.11)	(-26.42)
*NSec*_*ln*	0.0224***	0.0180***	0.0107***	0.00828***	0.0267***	0.0216***	0.0131***	0.0106***
	(59.83)	(51.73)	(24.67)	(20.34)	(68.55)	(59.80)	(29.64)	(25.62)
*Constant*	0.0854***	0.0698***	0.107***	0.0831***	0.101***	0.0812***	0.124***	0.0943***
	(37.45)	(32.96)	(41.05)	(31.93)	(43.45)	(37.17)	(46.64)	(35.25)
N	128960	109412	128960	109412	128960	109412	128960	109412
*R* ^2^	0.071	0.099	0.034	0.028	0.094	0.126	0.045	0.036

Note: t value is shown in brackets

* * *** and * represent significant at 1%, 5% and 10%, respectively.

Columns (2) (4) (6) and (8) show that a wealthy individual investor’s investment return is significantly positive relate to its correlated wealthy individual investors’ return, indicating that the trading behavior of a wealthy individual investor is affected by other connected investors in the network. Combined with the estimated coefficients in Table 6, it can be seen that when the variables of the rate of return of correlated wealthy individual investors are added, the influence direction and significance of variables such as the centrality of wealthy individual investors and transaction amount remain unchanged. Except that the regression coefficient of the centrality of eigenvectors decreases, the regression coefficient of each parameter changes little. This result shows that the influence of wealthy individual investors’ centrality on their return can be directly explained by the return of correlated investors to a great extent. Taking the arithmetic average return as an example, when changing the rate of return of one investor by 1 unit, the overall rate of return of other wealthy individual investors will change by 1.00307 units.

In addition, the trading times and transaction amount are negatively and significantly related to the rate of return of the wealthy individual investors, indicating that the return of the wealthy individual investors is limited by its scale. The larger the scale, the lower the rate of return. The quantity of companies that wealthy individual investors invested in is positively correlated with the return, which showing the investment diversity could also increase wealthy investors’ return.

Table 7 shows the regulation effect of wealthy individual investors’ average centrality on the impact of network density on stocks’ tail risk. The coefficient of the cross term (*dStkdense*×*Vecdu*) is significantly positive, indicating that a company’s network density of wealthy investors has a stronger influence on the stocks’ tail risk when the wealthy investors’ average centrality is higher. Besides, if the cross term is not taken in the regression process, network density (*dStkdense*) and investors’ average centrality (*Vecdu*) are regressed respectively, the results show the coefficients are both significantly positive to tail risks.

**Table 7:** Impact of wealthy individual investors’ behavior on stock prices’ tail risk.

Variables	Daily tail risks			Weekly tail risk		
	OLS	LOGIT	LOGIT	OLS	OLS	OLS
	(1)	(2)	(3)	(1)	(2)	(3)
	*dVol_Ampli*	*dVol_Upret*	*dVol_Downret*	*wVol_Ampli*	*wVol_Upret*	*wVol_Downret*
*dStkdense*×*Vecdu*	1.2203***	147.4430***	123.5179***	1.9531***	120.2074***	75.4848***
	(10.03)	(5.34)	(3.69)	(8.22)	(7.35)	(5.96)
*dStkdense*	0.0014	-0.0090	-0.7566***	-0.0061***	-0.2715**	-0.3004***
	(1.49)	(-0.04)	(-2.77)	(-3.16)	(-2.08)	(-2.97)
*Vecdu*	-0.7634***	-105.0496***	-70.4757***	-0.8378***	-58.4341***	-36.4400***
	(-8.94)	(-5.18)	(-2.92)	(-7.85)	(-8.22)	(-6.45)
*dHbigcnt*	0.0039***	0.8286***	0.8852***	0.0030***	0.1516***	0.1579***
	(35.28)	(40.61)	(36.35)	(16.45)	(15.20)	(19.81)
*dVol_t0*	0.3286***	1.0389***	1.1063***	0.4010***	0.2647***	0.1862***
	(77.89)	(36.42)	(29.76)	(43.49)	(26.56)	(17.30)
*Rm_Rf*	-0.4422***	-25.8505***	-98.5938***	-0.0914***	-3.0461***	-7.3131***
	(-45.25)	(-12.88)	(-35.38)	(-15.93)	(-7.60)	(-21.83)
*Amihud*	0.0161***	7.4449***	12.0442***	0.0099	1.6956***	1.5262***
	(5.70)	(17.42)	(24.74)	(1.46)	(4.26)	(4.68)
*Lnsize*	-0.0038***	-0.7915***	-0.8357***	-0.0031***	-0.1680***	-0.1537***
	(-42.99)	(-37.57)	(-32.97)	(-23.26)	(-19.74)	(-22.17)
*Bm*	-0.0022***	-0.5424***	-0.5153***	-0.0018***	-0.1404***	-0.1093***
	(-13.62)	(-13.69)	(-10.58)	(-7.57)	(-8.86)	(-8.35)
*Price*	0.0009***	-0.1470***	-0.0226	0.0012***	-0.0004	0.0265***
	(7.58)	(-5.24)	(-0.66)	(6.46)	(-0.03)	(2.64)
*Age*	0.0008***	0.1754***	0.1888***	0.0007***	0.0376***	0.0311***
	(7.40)	(7.93)	(7.05)	(4.60)	(3.84)	(3.93)
*Lev*	0.0006	-0.0936	-0.0606	0.0007	-0.0209	-0.0191
	(1.39)	(-1.13)	(-0.61)	(1.11)	(-0.51)	(-0.57)
*Roa*	-0.0154***	-2.8909***	-3.1570***	-0.0149***	-1.0796***	-0.9190***
	(-10.47)	(-10.05)	(-9.21)	(-7.18)	(-7.42)	(-7.74)
*Ac*	0.0032***	-0.2526	-0.2081	0.0017	-0.0223	0.0222
	(2.77)	(-1.02)	(-0.70)	(1.05)	(-0.21)	(0.25)
*Er*	-0.0005	0.3598**	0.3257*	0.0005	0.1126*	0.0682
	(-0.68)	(2.30)	(1.73)	(0.55)	(1.74)	(1.27)
*Rtr*	0.0019	-0.1851	0.1144	0.0047	0.5267	0.5385
	(0.45)	(-0.23)	(0.12)	(0.74)	(1.23)	(1.47)
*Oiebt*	-0.0002***	-0.0240***	-0.0239***	-0.0001***	-0.0135***	-0.0093***
	(-5.68)	(-5.10)	(-4.12)	(-3.14)	(-3.70)	(-3.29)
*Sum10*	0.0078***	2.1131***	2.1075***	0.0071***	0.5384***	0.4896***
	(16.22)	(19.37)	(15.89)	(9.97)	(11.40)	(12.91)
*Dis12*	-0.0000	-0.1719**	-0.1512*	-0.0002	-0.0805***	-0.0523**
	(-0.03)	(-2.39)	(-1.72)	(-0.39)	(-2.61)	(-2.13)
*Constant*	0.0821***	11.3753***	11.5643***	0.0688***	3.2860***	2.8291***
	(42.69)	(24.14)	(20.39)	(23.90)	(17.63)	(18.41)
Industry FE	yes	yes	yes	yes	yes	yes
N	113,347	113,347	113,347	24,088	24,088	24,088
*R*^2^*(*Pseudo *R*^2^*)*	0.251	0.1348	0.1669	0.380	0.198	0.171

Note: *dVol_t0* is the lag term of the corresponding daily fluctuation, that is, *dVol_Ampli_t0、dVol_Upret_t0、dVol_Downret_t0* respectively. Data in parentheses is the t value

***, ** and * indicate significant at the 1%, 5% and 10% levels, respectively. The last data in Column (1) is *R*^2^ and in Column (2) (3) are Pseudo *R*^2^.

In the robustness test, the sample interval is divided into two parts. The wealthy individual investors’ account data from January to April in 2017 are used to construct the network to analyze their network characteristics and impacts from May to June. Results show that (1) according to the historical transaction resonance, wealthy individual investors will still resonate in the future and form transaction resonance; (2) Wealthy individual investors can earn more by increasing their centrality in the network; (3) Compared with edge investors, central investors in stock enhance the influence of investors’ network density on stocks’ tail risk. As a result, The wealthy individual investors’ network exists and has behavior spillover effect, and wealthy individual investors have the motivation to affect the trading decisions of other investors, leading to abnormal price volatility.

## 6. Further analysis

### 6.1 Portfolio constructed based on wealthy individual investors’ network density

According to the daily and weekly network density of wealthy individual investors, this paper divides all stocks into ten groups and constructs the portfolio of buying the highest density group and selling the lowest group to analyze whether this trading strategy can bring an excess return to investors. First, calculate the daily or weekly network density of each stock, and sort the stocks according to the lagged value of the network density, and then divide the overall sample into 10 groups as 10 portfolios. Second, a zero-cost portfolio “10–1” of stock assets is constructed, that is, buying Portfolio 10 with high network density and selling Portfolio 1 with low network density. Third, the average rates of return of these above 11 portfolios are calculated, and the Fama-French three-factor model is applied to the portfolio’s rate of return for risk adjustment. According to classical asset pricing theory, risk-adjusted intercept terms reflect an excess rate of return that cannot be explained by risk factors.

$$r_{pt}-r_{ft}=\alpha_{1}+\beta_{1}(r_{Mt}-r_{ft})+\beta_{2}{SMB}_{t}+\beta_{3}{HML}_{t}+\varepsilon_{t}$$ (10)

Where, *r*_*pt*_ represents the average return of the stock portfolio in the period *t*, *r*_*ft*_ represents the risk-free rate of return in the period *t*, *r*_*Mt*_ represents the market’s rate of return in the period *t*, and *SMB*_*t*_ and *HML*_*t*_ represent the size factor and book-to-market ratio factor proposed by Fama and French (1993) [43] respectively.

**Fig 1:**
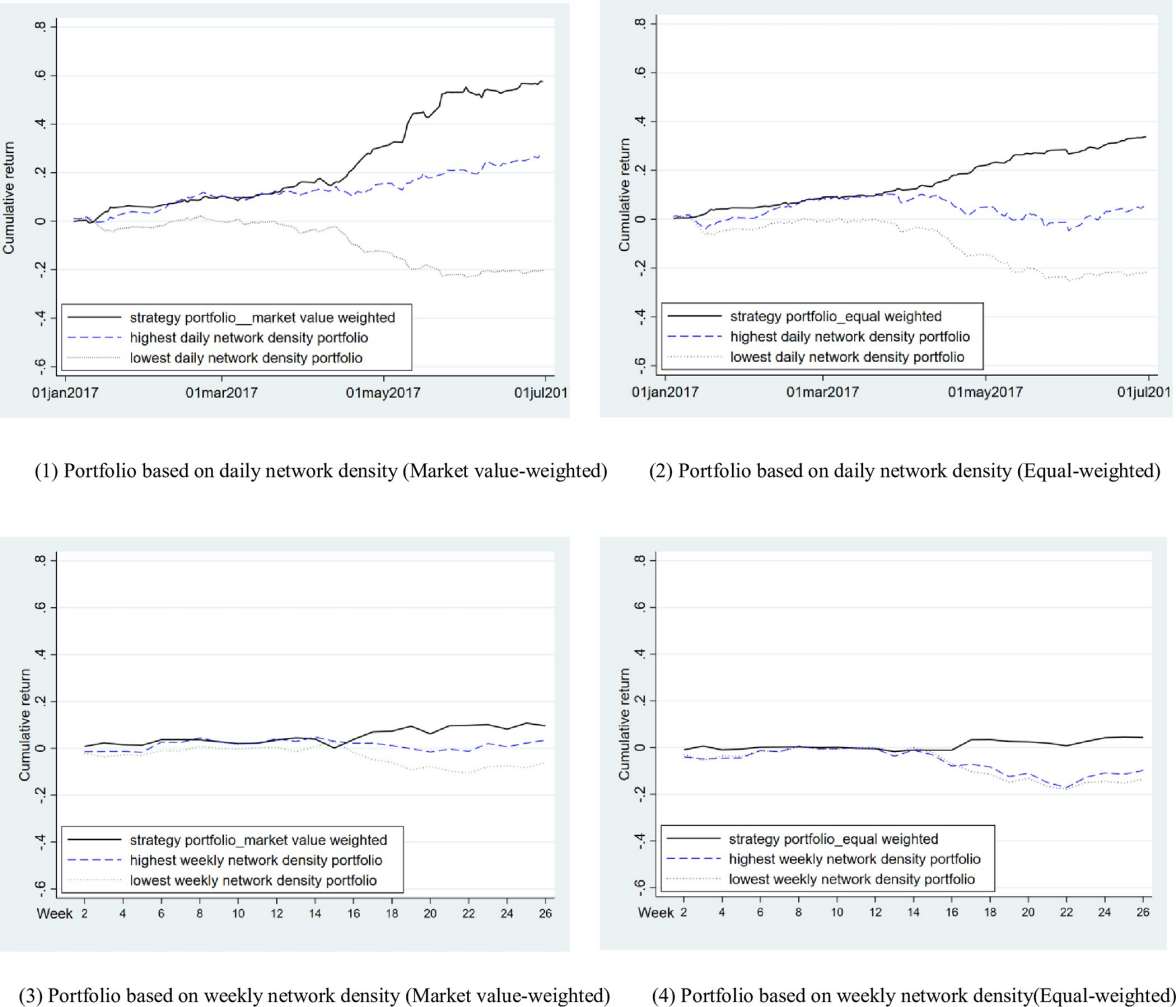
Cumulative return of a strategy portfolio based on wealthy individual investors’ network density. (1) Portfolio based on daily network density (Market value-weighted). (2) Portfolio based on daily network density (Equal-weighted). (3) Portfolio based on weekly network density (Market value-weighted). (4) Portfolio based on weekly network density(Equal-weighted).

Table 8 shows the excess return of portfolios based on wealthy individual investors’ network density. The results in columns (1)—(2) of Table 8 show that the higher the daily network density of the portfolio, the higher the excess return obtained after risk adjustment. The excess return of the stock portfolio with high network density is significantly positive, and that with low network density group is significantly negative. Portfolio 10 with the highest network density has a daily excess rate of return of 0.13% and the annualized rate of return of 32.5% (= 0.13%×250), which means that if investors adjust the portfolio every day, buying the stocks with the top 10% of the daily network density, they can obtain an annual risk-adjusted excess rate of return of 32.5%. The zero-cost “10–1” portfolio reaches 0.26% of the daily excess rate of return and is significant at 1%, indicating that the daily excess rate of return of 0.26% could be achieved by buying the stock portfolio with the highest network density and selling the stock portfolio with the lowest network density. Fig 1 demonstrates that the cumulative return from the strategy portfolio is higher than the return from the portfolio with the highest and the lowest network density.

**Table 8:** Analysis of excess rate of return of wealthy individual investors based on network density.

Stock Dense grouping	Daily portfolio				Weekly portfolio			
	(1)		(2)		(3)		(4)	
	Market value weighted return		Equal weighted return		Market value weighted return		Equal weighted return	
	Alpha	R^2^	Alpha	R^2^	Alpha	R^2^	Alpha	R^2^
(1) lowest	-0.0016***	0.78	-0.0013***	0.94	0.0009	0.737	-0.0006	0.932
	(-4.00)		(-5.99)		(0.38)		(-0.43)	
(2)	-0.0010***	0.84	-0.0013***	0.959	0	0.796	-0.0014	0.945
	(-3.33)		(-6.88)		(-0.01)		(-0.97)	
(3)	-0.0007**	0.786	-0.0010***	0.944	0.001	0.747	-0.0003	0.932
	(-2.60)		(-4.57)		(0.64)		(-0.21)	
(4)	-0.0005	0.761	-0.0005**	0.947	0.0009	0.852	-0.0006	0.966
	(-1.62)		(-2.23)		(0.63)		(-0.66)	
(5)	-0.0007**	0.731	-0.0006***	0.929	0.0004	0.698	-0.0025*	0.906
	(-2.46)		(-2.79)		(0.22)		(-1.75)	
(6)	0.0000	0.679	-0.0003	0.941	-0.0008	0.842	-0.0017	0.954
	(-0.11)		(-1.23)		(-0.63)		(-1.52)	
(7)	-0.0001	0.663	0.0002	0.92	0.0001	0.58	0.0004	0.940
	(-0.35)		(0.65)		(0.03)		(0.34)	
(8)	0.0002	0.737	0.0003	0.906	0.0001	0.902	-0.0006	0.936
	(0.65)		(1.05)		(0.08)		(-0.46)	
(9)	0.0011***	0.724	0.0011***	0.931	0.0019	0.766	0.0006	0.943
	(2.80)		(3.97)		(1.10)		(0.43)	
(10) highest	0.0013***	0.573	0.0012***	0.926	-0.0005	0.726	0.0024	0.911
	(2.72)		(5.14)		(-0.23)		(1.21)	
(10)-(1) strategy	0.0026***	0.352	0.0024***	0.022	-0.0015	0.441	0.0029	0.075
	(3.93)		(7.56)		(-0.48)		(1.02)	

This paper calculates the weekly network density of wealthy individual investors and constructs the portfolio according to the number of wealthy individual investors and their correlation. As is shown in columns (3)—(4) of Table 8, the portfolio based on the weekly network density does not obtain an excess return, which indicates that wealthy individual investors’ trading resonance do not last a long time, and the impact of wealthy individual investors’ transactions on individual stocks changes rapidly.

This result is consistent with the conclusions of Shi (2017) [4], which proposed that some investors take the capital advantage to affect short-term trends of stock prices and plunder the subsequent followers, which is why they hold stocks for only 1–5 days and shows the behavior of fast in and fast out. This paper finds that wealthy individual investors have a significant impact on stock prices through trading resonance rather than personal trading behavior. Besides, wealthy investors’ trading resonance is effective in raising daily stock prices, but does not significantly affect weekly stock prices.

### 6.2 Portfolio based on wealthy individual investors’ net network centrality

This paper further constructs the portfolio based on the daily average centrality of wealthy individual investors in individual stocks. The calculation method of stock portfolio’s excessive rate of return based on network density is consistent with that of stock portfolio based on network centrality. The average eigenvector centrality of wealthy individual investors calculated in this paper is based on the data at the end of the previous period to avoid endogeneity problems. In addition, this paper examines the impact of the weekly average eigenvector centrality of the stock portfolio on the rate of return.

**Fig 2:**
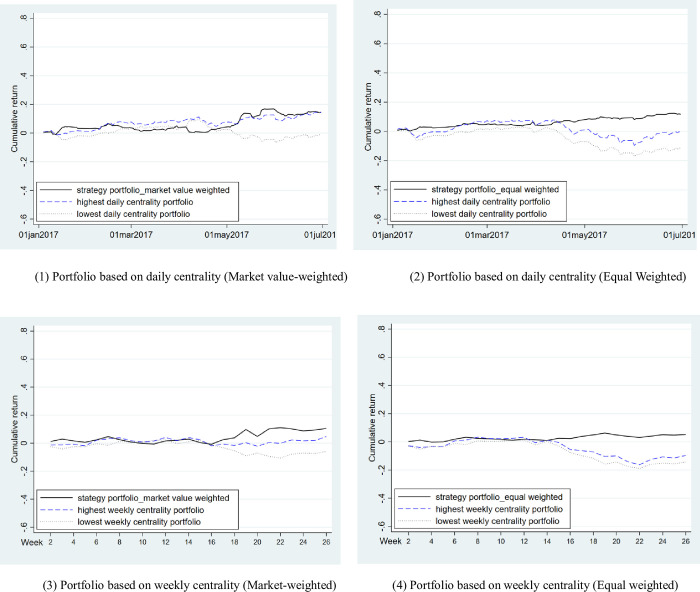
Cumulative return of the strategy portfolio based on wealthy individual investors’ network centrality. (1) Portfolio based on daily centrality (Market value-weighted). (2) Portfolio based on daily centrality (Equal Weighted). (3) Portfolio based on weekly centrality (Market-weighted). (4) Portfolio based on weekly centrality (Equal weighted).

Table 9 shows the excess return of portfolios based on the network centrality of wealthy individual investors. The strategy portfolio based on the daily or weekly network centrality of wealthy individual investors does not obtain the excess return adjusted by Fama-French three factors. The excess return of portfolios both on a daily basis and on a weekly basis does not increase with the increase in group numbers. The cumulative return of the strategy portfolio based on the network centrality of wealthy individual investors is shown in Fig 2. The strategy portfolio does not consistently obtain an excess return, implying that the influence of wealthy individual investors on stock return mainly comes from their trading resonance and trading behavior without trading resonance does not significantly affect stock return even if a large number of wealthy individual investors with high centrality are gathered.

**Table 9:** Analysis of excess return the portfolio based on wealthy individual investors’ network centrality.

Eigenvector centrality grouping	Daily portfolio				Weekly portfolio			
	(1)		(2)		(3)		(4)	
	Market value weighted return		Equal weighted return		Market value weighted return		Equal weighted return	
	Alpha	R^2^	Alpha	R^2^	Alpha	R^2^	Alpha	R^2^
(1) lowest	0.0003	0.69	0.0004*	0.946	0.0001	0.865	0.0018	0.959
	(0.74)		(1.72)		(0.04)		(1.30)	
(2)	0.0007*	0.752	-0.0001	0.956	0.0006	0.724	-0.0009	0.931
	(1.86)		(-0.27)		(0.31)		(-0.55)	
(3)	-0.0002	0.734	-0.0005*	0.943	0.0008	0.66	-0.0003	0.959
	(-0.45)		(-1.91)		(0.41)		(-0.25)	
(4)	-0.0004	0.798	-0.0004**	0.946	0.0025	0.806	-0.002	0.948
	(-1.20)		(-2.13)		(1.27)		(-1.56)	
(5)	-0.0001	0.701	-0.0003	0.942	-0.0004	0.717	-0.0002	0.967
	(-0.41)		(-1.61)		(-0.21)		(-0.23)	
(6)	-0.0003	0.754	-0.0007***	0.955	0	0.775	-0.0019	0.947
	(-0.82)		(-3.35)		(-0.02)		(-1.59)	
(7)	-0.0001	0.628	-0.0004	0.931	0.0008	0.871	-0.0003	0.955
	(-0.20)		(-1.56)		(0.60)		(-0.33)	
(8)	-0.0001	0.692	-0.0005**	0.935	0.0008	0.687	-0.0002	0.884
	(-0.21)		(-2.35)		(0.42)		(-0.12)	
(9)	-0.0003	0.646	-0.0002	0.913	0.0005	0.867	-0.0005	0.929
	(-0.90)		(-0.90)		(0.41)		(-0.46)	
(10) highest	0.0009***	0.69	0.0005**	0.921	0.0001	0.444	0.0002	0.931
	(2.69)		(2.03)		(0.07)		(0.16)	
(10)-(1) strategy	0.0004	0.044	-0.0001	0.209	-0.0001	0.164	-0.0017	0.171
	(0.83)		(-0.35)		(-0.02)		(-1.19)	

## 7. Conclusions

This paper utilizes the account data of 128,960 individual investors with a market value of more than CNY 10 million to study the impact of wealthy individual investors’ behavior on the tail risk of the stock market from the perspective of trading networks. By defining pairwise investors with similar trading behavior as connected investors, this paper constructs a wealthy individual investors’ overall network and proposes a definition of network density for stocks’ sub-networks. Results show that the tail risk of stock prices is positively correlated with the network density of wealthy individual investors, indicating that the similar transaction action of wealthy individual investors is one of the reasons for the tail risk of the stock market. In the endogenous test, to avoid the potential possibility that wealthy individual investors choose high fluctuating stocks to invest on purpose, this paper uses the historical transaction record of wealthy individual investors in the lag period to extract the trading resonance and establish investors’ relationship. Endogenous tests’ results support the paper’s findings and show that the renewed investors networks do explain the subsequent stock return and stock fluctuations.

This paper uses a spatial econometric model to investigate the behavior mechanism of wealthy individual investors’ network density affecting stocks’ tail risk. Results show that wealthy individual investors are affected by the connected investors in the network and conduct concurrent transactions, and the behavior influence mechanism of wealthy individual investors lies in the spillover effect of their trading decisions in the networks. By the social network analysis method, this paper also discovers that wealthy individual investors can profit from their location in the network which further supports the behavior motivation of wealthy individual investors’ concurrent transactions.

This paper further discovers that the average centrality of stocks’ wealthy investors strengthens network density’s affection on stocks’ tail risk, indicating that more central investors have more influence power on other investors and thus on the financial markets. Besides, the portfolio based on the daily network density of wealthy individual investors can obtain a significant excess return, while other portfolio based on investors’ network average centrality or different period of time do not gain an excess return, suggesting that the strategy of following wealthy individual investors to invest in popular stocks is effective but it does not last long.

From the theory and practical perspectives, the research results of this paper provide not only micro evidence for the formation mechanism of asset prices, but also a theoretical reference for effectively intervening in the tail risk of the stock market caused by wealthy individual investors’ transactions resonance.
