# Correction: Wealthy individual investors and stock markets’ tail risk

**DOI:** 10.1371/journal.pone.0317285

**Published:** 2025-01-06

**Authors:** The PLOS One Staff

The first author’s name is spelled incorrectly. The correct name is: Yu He. The correct citation is: He Y, Lu R, Yang H, Zhang B (2024) Wealthy individual investors and stock markets’ tail risk. PLOS ONE 19(5): e0282173. https://doi.org/10.1371/journal.pone.0282173

There are errors in the Funding statement. The correct Funding statement is as follows: Project funded by National Natural Science Foundation of China (Grant No. 72173081, No. 71773072, and No. 72073088), China Postdoctoral Science Foundation (Grant No. 2018M630420) and Soft Science Research Project of Zhejiang Province in China (Grant No. 2019C25022).

The publisher apologizes for the errors.
